# Immunological Linkages Between Inflammatory Bowel Diseases and Type 2 Diabetes

**DOI:** 10.3390/biomedicines13092224

**Published:** 2025-09-10

**Authors:** Davide Frumento, Ștefan Țălu

**Affiliations:** 1 Department of Pharmacy, University of Genoa, Viale Benedetto XV 7, 16132 Genoa, Italy; 2 The Directorate of Research, Development and Innovation Management (DMCDI), The Technical University of Cluj-Napoca, Constantin Daicoviciu Street, No. 15, 400020 Cluj-Napoca, Romania

**Keywords:** IBD, diabetes, endocrinology, gastroenterology, epidemiology

## Abstract

**Background:** Inflammatory bowel disease (IBDs) are chronic, immune-mediated disorders of the gastrointestinal tract, encompassing ulcerative colitis (UC) and Crohn’s disease (CD), and primarily affecting individuals with genetic susceptibility. IBDs are characterized by dysregulated mucosal immune responses to intestinal microbiota, leading to sustained inflammation and tissue damage. These conditions not only pose a significant burden on healthcare systems but are also frequently associated with distinct comorbidities. **Rationale:** Given the immunological nature of both IBDs and type 2 diabetes (T2D)—each involving a complex interplay between genetic predisposition and environmental triggers—an increasing number of studies have suggested a pathophysiological link between the two. Both diseases involve chronic low-grade inflammation and alterations in immune signaling pathways, such as cytokine dysregulation, T-cell imbalance, and aberrant innate immune activation. **Methods:** To investigate this association more robustly, we conducted a cohort study involving 49 consecutive patients diagnosed with both IBD and T2D. **Results:** Our findings revealed a strong correlation between the two conditions, with UC emerging as the predominant IBD subtype linked to T2D. Notably, the highest prevalence was observed in patients aged 65–74 years, suggesting age-related immune modulation may play a role. In a matched case-control analysis (48 cases vs. 96 controls), 70.8% of the IBD–T2D cases were diagnosed with UC, 25.0% with CD, and 4.2% with indeterminate colitis. Similarly, in the cohort study, UC accounted for 73.81% of cases, CD for 21.43%, and non-determined colitis for 4.76%. **Conclusions:** These data support the hypothesis that UC, more so than CD, exhibits a stronger immunological and clinical association with T2D. Interestingly, CD was absent in the 55–64 age group, potentially indicating age-specific immunological trajectories or differential environmental exposures. The observed patterns reinforce the concept that immune dysregulation is a shared underpinning of both IBD and T2D, and that UC may serve as an immunological bridge linking gastrointestinal and metabolic inflammation.

## 1. Introduction

Ulcerative colitis (UC) and Crohn’s disease (CD) are the most significant conditions within the spectrum of inflammatory bowel diseases (IBDs), characterized by chronic immune-mediated inflammation of the gastrointestinal tract. These diseases predominantly affect genetically predisposed individuals, whose immune systems fail to tolerate the commensal microbiota, leading to chronic inflammation and mucosal damage. Such immunological dysfunctions underlie the pathogenesis of both UC and CD, involving the dysregulation of T-cell responses, cytokine production, and innate immune signaling [1].

IBDs are high-cost diseases, not only due to direct medical expenses but also because of their specific morbidity and co-morbidity profiles. The immunological nature of these diseases contributes to various extra-intestinal manifestations, which significantly impact patients’ quality of life, particularly due to the need for surgeries and management of complications [2]. UC and CD are recognized as global health challenges, with an incidence rate of 12.7 and 24.3 cases per 100,000 people per year in Europe, respectively, and a prevalence of 0.5% and 1.0%. Their incidence is rising worldwide, affecting both adults and children [3].

Co-morbidities in IBD patients often include other immune-mediated diseases such as asthma, psoriasis, type 1 diabetes (T1D), rheumatoid arthritis, multiple sclerosis, systemic lupus erythematosus, vitiligo, autoimmune thyroid disease, and chronic glomerulonephritis [4]. T1D is particularly common, and its association with IBD is of great clinical importance. This association is believed to be rooted in shared immunological dysfunctions, such as defects in immune tolerance and autoimmunity. T1D has also been directly linked to a dysfunctional intestinal immune response, which disrupts bowel homeostasis, a factor that contributes to the pathogenesis of IBD. Interestingly, there is no study that has assessed the prevalence of type 2 diabetes (T2D) in IBD patients [5,6].

Among IBD subtypes, UC has been shown to have a higher prevalence of T1D, particularly in children, compared to CD, highlighting potential differences in the immune pathways driving both diseases [7]. Moreover, IBD patients receiving immunosuppressive therapies are at a significantly higher risk for infections and surgical complications, which may be exacerbated by immune dysregulation [8,9]. While immunosuppressive therapies play a crucial role in managing IBD, their associated risks, such as increased susceptibility to infections and surgical complications, highlight the complex nature of disease management and the broader implications of immune dysregulation in IBD. This dysregulation, in turn, is influenced by both genetic and environmental factors, which contribute to the pathogenesis of the disease.

The pathogenesis of IBD is multifactorial, with genetic and environmental factors playing pivotal roles. IBDs are known to occur more frequently in individuals carrying certain genetic risk alleles (i.e., those associated with immune system regulation) which may increase susceptibility to the disease, though these genetic factors alone are not sufficient to cause it. Studies have proposed a genetic model wherein UC and CD are related conditions, sharing some susceptibility loci but differing in others, which contributes to the divergent disease progression and clinical manifestations [10,11]. Environmental factors also play a key role in triggering IBDs in genetically predisposed individuals, with pharmacological agents such as antibiotics, isotretinoin, etanercept, rituximab, ipilimumab, oral contraceptives, and nonsteroidal anti-inflammatory drugs (NSAIDs) being considered potential disease triggers [12]. In particular, NSAIDs have been shown to induce acute colon ulcers with histological features of CD, accompanied by sideropenic anemia, diarrhea, and abdominal pain [13].

Corticosteroids remain a cornerstone in IBD treatment, particularly in controlling moderate to severe flare-ups. These drugs are administered via various routes (oral, topical, intravenous) and provide rapid symptom relief. However, corticosteroids are ineffective in maintaining remission and are associated with numerous adverse effects, including metabolic disturbances such as insulin resistance, which may increase the risk of developing T2D. A better understanding of corticosteroid mechanisms has led to the development of novel, less toxic corticosteroids with fewer side effects. The insulin resistance induced by corticosteroids in IBD patients can lead to elevated blood glucose levels, which, over time, may increase the risk of developing type 2 diabetes. This metabolic disruption is clinically significant, as it may contribute to higher morbidity and mortality rates among IBD patients, particularly those with long-term steroid use [14].

The metabolic disturbances induced by corticosteroids and other immunosuppressive therapies, including insulin resistance and alterations in lipid metabolism, may exacerbate the risk of developing type 2 diabetes (T2D) in patients with inflammatory bowel disease (IBD). These metabolic disruptions not only contribute to the progression of comorbidities such as T2D but could also influence the severity of IBD complications, leading to a worse overall prognosis. This underscores the importance of understanding the interplay between IBD treatments, metabolic dysfunction, and long-term patient outcomes, justifying the aim of investigating these connections further [14].

CD-related mortality is reported to be higher than that of the general population, with younger ages at diagnosis and the need for multiple surgeries identified as additional risk factors. Despite advancements in clinical treatment, the management strategies for CD have not significantly improved over the last three decades, and improvements in survival remain limited. CD is also associated with mortality due to both malignant and non-malignant gastrointestinal disorders, excluding colorectal cancer. Approximately 30% of deaths in CD patients are directly related to the disease. In contrast, UC-related mortality is generally lower, although surgery is associated with increased mortality rates. Disease extent and age at diagnosis do not significantly correlate with survival in UC, unlike in CD. However, an increased risk of mortality within the first year after diagnosis has been noted, possibly due to acute severe colitis [15].

The potential causality between type 2 diabetes (T2D) and inflammatory bowel diseases (IBD) is suggested due to several shared mechanisms, though definitive evidence remains scarce. Chronic inflammation is a common feature of both conditions, with inflammatory cytokines like tumor necrosis factor-alpha (TNF-α) and interleukin-6 (IL-6) promoting insulin resistance, a hallmark of T2D. In IBD, dysregulated immune responses in the gut may exacerbate systemic inflammation, potentially leading to metabolic disturbances. Additionally, alterations in the gut microbiome, a key factor in both diseases, may contribute to the pathogenesis of both conditions through immune modulation and metabolic dysfunction. Epidemiologic studies have indicated a higher prevalence of metabolic disorders in IBD patients, and some studies have observed an increased risk of T2D in these individuals, though causality remains unclear. A plausible hypothesis is that the persistent inflammatory environment in IBD could contribute to insulin resistance over time, thus increasing the likelihood of T2D development.

The aim of this study is to determine the prevalence of T2D in CD and UC patients and to evaluate whether the coexistence of these diseases is associated with an increased risk of abdominal complications and a negative prognosis for the intestinal disease. The relevance of this investigation lies in the potential causality between T2D and IBDs, which is yet to be definitively established, but could lead to new primary prevention strategies aimed at reducing the incidence of both conditions.

This study is important because it sheds light on the emerging immunological and clinical connection between inflammatory bowel diseases (particularly ulcerative colitis) and type 2 diabetes. By demonstrating a strong correlation—especially among older adults—it highlights the potential for shared inflammatory and immune mechanisms driving both conditions. The findings not only deepen our understanding of the pathophysiology linking gastrointestinal and metabolic diseases but also underscore the need for integrated approaches to managing comorbid chronic illnesses.

## 2. Materials and Methods

### 2.1. Case–Control Study

A preliminary case-control study was conducted. A total of 144 patients, 48 cases compared to 96 controls (cases: IBD + diabetes and controls: IBD only), were consecutively selected, specifically in instances of comorbidity with diabetes mellitus (both type 1 and type 2) (Table 1).

To qualify for inclusion, patients needed to be diagnosed with both IBDs (ulcerative colitis and/or Crohn’s disease) and diabetes mellitus. Patients with IBDs who did not have diabetes mellitus were excluded from this study. The analysis was executed through a database examination. Each case was matched with two controls who had IBDs but did not have a diabetes diagnosis or hyperglycemia. We matched cases (IBD + diabetes) and controls (IBD only) 1:1 based on key covariates including age, sex, disease type, disease severity, and treatment regimen, using the nearest-neighbor method without replacement to ensure balanced groups for comparison. In both the case and control groups, the use of immunosuppressive or biological therapy, as well as surgical interventions, were evaluated as prognostic indicators.

T2D was defined employing ADA (American Diabetes Association) diagnostic criteria: A1C level of 6.5% or higher, a fasting plasma glucose (FPG) level of 126 mg/dL (7.0 mmol/L) or higher, a two-hour plasma glucose level of 200 mg/dL (11.1 mmol/L) or higher during an oral glucose tolerance test (OGTT), or a random plasma glucose level of 200 mg/dL (11.1 mmol/L) or higher in a patient exhibiting classic symptoms of hyperglycemia or a hyperglycemic crisis. In cases without clear evidence of hyperglycemia, diagnosis requires confirmation through repeat testing on a different day or using an alternative abnormal test.

### 2.2. Patient Cohort and Eligibility Criteria

In order to obtain a more precise understanding of the correlation between inflammatory bowel disease (IBD) and diabetes, a cohort study was conducted involving 49 consecutive patients who were diagnosed with both IBDs and type 2 diabetes (T2D). The eligibility and exclusion criteria mirrored those of a case-control study. Patients with diabetes resulting from both steroid use and other causes, such as pancreatic resection and haemochromatosis, were excluded to eliminate potential confounding variables.

### 2.3. Statistical Analysis

Quantitative data are expressed as the mean values ± standard deviation. Differences in frequency were assessed using a χ^2^ test, and for smaller sample sizes, a *t*-test was employed to compare means. A *p*-value of less than 0.05 was deemed to indicate statistical significance. The prevalence of diabetes mellitus (DM) in the evaluated population was compared with the ISTAT report from 2010–2011 based on case-control data. Genetic variability was identified as a potential confounding factor, albeit one that could not be avoided. The data were sourced from Milan University Hospital, Italy, in 2017. This research constituted an epidemiological and biostatistical study, with calculations performed using SPSS software (v31). To mitigate potential biases, patients were selected consecutively, thereby eliminating the possibility of prejudice and errors. The minimum sample size for the case-control study was determined using an online tool (https://www.surveysystem.com/sscalc.htm, accessed on 10 August 2025) to be at least 142 (our sample comprised 144), while the minimum requirement for the cohort study was 49 (our figure was precisely 49). No cases needed to be excluded, as there were no missing data. The confidence level was 95%, while the desired statistical power was 80% and, with this being an observational study, there were no expected proportions (the effect size considered meaningful was 0.2, as per Pearson’s metrics for small effects).

Genetic variation and environmental factors are well-known confounders in many studies, particularly those examining complex traits or health outcomes. In this study, while the authors briefly mention these factors as potential confounders, they do not provide sufficient information on how these variables were controlled for in the analysis, which is a critical oversight. Genetic differences among individuals can significantly influence the outcomes being studied. Variations in genes related to disease susceptibility, metabolism, or other physiological processes may confound the relationships being investigated. If not accounted for, genetic factors could lead to misinterpretations of the true effect of the exposures or treatments under study. For example, if one group has a higher proportion of individuals with a genetic predisposition to a certain condition, the results might be skewed, and the observed effects could be attributed to genetic factors rather than the primary variable of interest. Environmental exposures—such as diet, lifestyle, pollution, or socioeconomic status—can also act as confounding factors. These variables may interact with genetic predispositions to influence health outcomes in ways that are not immediately apparent. For example, if individuals in one group are more likely to live in a polluted area, the environmental factor could mask or exaggerate the true relationship between the studied variables. Failure to control for such factors can lead to biased conclusions about the primary association being studied.

To minimize the impact of genetic differences, participants were selected to represent a relatively homogeneous genetic background, and random assignment was employed to ensure that any residual genetic variability was evenly distributed across experimental groups. The timing and location of data collection were kept consistent across all experimental sessions. These combined strategies strengthen the internal validity of the findings by reducing the likelihood that observed effects are attributable to uncontrolled genetic or environmental differences rather than the experimental manipulation itself.

While increasing sample size and including diverse populations can enhance generalizability, this is not aligned with this study’s purpose. This study is designed to explore a phenomenon within a specific population and context, expanding beyond that scope can introduce confounding variables and dilute this study’s focus. A defined population helps ensure internal validity and provides a foundation for broader studies in the future.

Using a control group twice the size of the experimental group (96 controls vs. 48 cases) enhances statistical power, reduces the risk of Type II errors, and improves this study’s ability to detect true associations. A larger control group helps minimize bias, accounts for individual variability, and ensures more accurate comparisons by better matching confounding factors like age and sex. This approach strengthens the validity and generalizability of the findings, making the results more reliable.

Some statistical results did not reach conventional levels of significance, which may be attributed to factors such as limited sample size, variability within the data, or the exploratory nature of this the study. While these findings should be interpreted with caution, they may still suggest meaningful trends that warrant further investigation in larger or more targeted studies.

## 3. Results

### 3.1. Case-Control Design and Age Stratification

**Figure 1:**
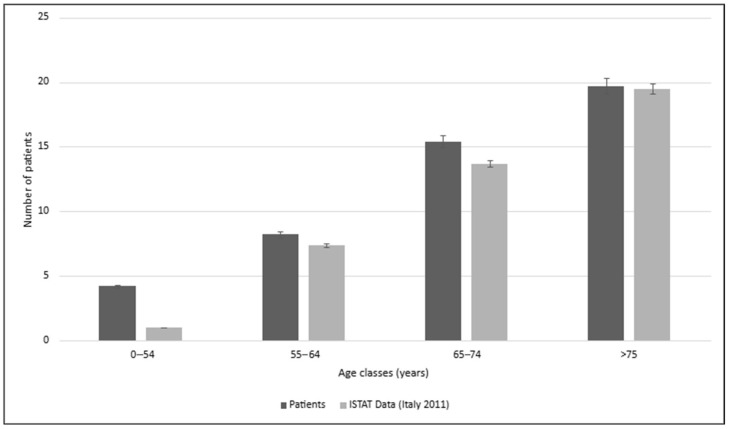
Prevalence of diabetes mellitus (both type 1 and 2) in IBD patients and ISTAT 2011 Italian data.

A sample of 48 IBD cases was compared with 96 controls, with a focus on immunological factors influencing the coexistence of diabetes mellitus (DM)—both type 1 (T1D) and type 2 (T2D). Subdividing the data by age groups reveals that IBD cases are associated with a higher prevalence of DM (Figure 1) compared to the 2011 ISTAT Italian data, particularly in the 0–54 years old range (*p* = 0.017) [16].

**Figure 2:**
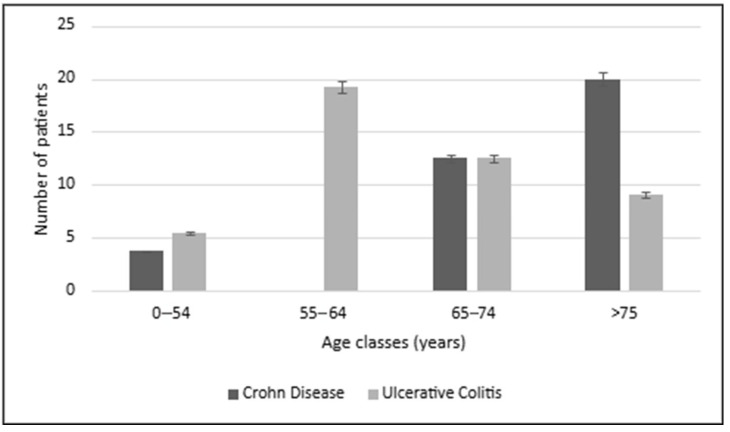
Diabetes mellitus (both type 1 and 2) distribution in IBD patients (case/control study).

This result could be influenced by the fact that IBDs typically manifest across a broad age range, from childhood to the sixth decade [16], while T2D generally develops later in life (mean age 50.3 ± 11.2 years, as reported by Noh et al.) [17]. In contrast, T1D typically presents before the age of 20 [18], which may shift the observed age range. From a disease distribution standpoint, ulcerative colitis (UC) appears to be more strongly linked with DM than Crohn’s disease (CD), especially in the 0–65 years old range (*p* < 0.05, Figure 2).

UC is primarily characterized by an overactive Th2 and Th17-mediated immune response, which may predispose patients to systemic inflammation and insulin resistance, common features of T2D. In contrast, CD, which is often associated with a more complex Th1/Th17-driven inflammatory response, exhibits a more heterogeneous immune profile that may not coincide as strongly with metabolic disturbances observed in DM. The sex distribution of the patient cohort showed a predominance of male patients (62.5%), suggesting a potential male sex-related immunological predisposition to both IBDs and DM. In IBDs, males are more likely to develop Crohn’s disease, and sex differences in immune responses, influenced by testosterone, may contribute to increased male susceptibility to inflammatory diseases [19]. Of the 48 patients with IBD, 18.7% were diagnosed with T1D, while 81.3% had T2D, indicating a stronger yet hypothetical correlation between IBDs and T2D. Interestingly, 70.8% of patients had UC, while 25.0% had CD, and the remaining 4.2% had undetermined colitis. This finding further emphasizes that UC, more than CD, appears to be more strongly associated with DM, though CD remains a related pathology (*p* < 0.05). Notably, CD was absent in the 55–64 years old range (Figure 2), which could reflect both the higher UC–DM correlation and the consecutive patient selection method, which may have biased the findings towards younger patients with more severe disease. Immunological treatments (Table 2) such as biological therapies and immunosuppressive drugs play a significant role in managing both IBDs and DM.

Interestingly, a significant proportion of both cases (27.1%) and controls (20.8%) used immunosuppressive therapies, which are known to modulate the immune response and could influence the clinical presentation of both conditions. A smaller percentage of patients used biological therapy (4.2% in cases and 13.5% in controls), which targets specific immune molecules like TNF-α, IL-12/23, or integrins. The effect of these therapies on the immune system may alter the disease course and potentially influence the co-occurrence of IBDs and DM. Surgical intervention was required for 20.8% of cases, compared to 7.2% of controls, suggesting that the coexistence of DM could increase the risk of requiring surgery. This aligns with previous observations that patients with IBD and concurrent DM may experience worse disease outcomes due to altered immune responses, delayed wound healing, and higher susceptibility to infections. Interestingly, the need for surgery typically arose after the onset of IBDs, suggesting that while diabetes is a manageable condition, IBDs, particularly when co-occurring with DM, may shift the disease trajectory towards more severe complications requiring surgical intervention (differences are descriptive only). Both Crohn’s disease (CD) and ulcerative colitis (UC) are associated with high mortality and surgical risks, emphasizing the need to explore factors that could further complicate patient outcomes, such as the development of type 2 diabetes (T2D). T2D is known to contribute to increased cardiovascular risk, impaired wound healing, and a heightened risk of infections, all of which could exacerbate the challenges of managing IBD. Additionally, the presence of T2D may interfere with the effectiveness of standard IBD treatments, as metabolic dysfunction can alter immune responses and influence drug metabolism. These factors could lead to poorer disease control, increased disease flares, and ultimately worse prognosis. In addition, overweight and obesity (39.6% and 16.7%, respectively) were observed in the cohort, suggesting a possible positive correlation between body mass index (BMI) and IBD. This association is intriguing, as obesity-related chronic inflammation is known to exacerbate both IBD and diabetes through immune activation, particularly involving the innate immune system and macrophage activation. Within the high BMI category, individuals classified as overweight (but not obese) were found to have a higher prevalence of IBD-DM coexistence, speculatively indicating that even moderate obesity could exacerbate the immune-mediated processes that drive both diseases. A genetic predisposition for DM was noted in 31.3% of the individuals studied, suggesting a potential genetic link between IBDs and diabetes. However, further research is needed to determine the precise genetic factors that predispose individuals to both conditions. The possibility that IBD and DM share some common susceptibility loci (e.g., NOD2 in IBD and various T2D-related loci) raises important questions about the immunogenetic pathways linking these diseases.

### 3.2. Age-Based Cohort Distribution

**Figure 3:**
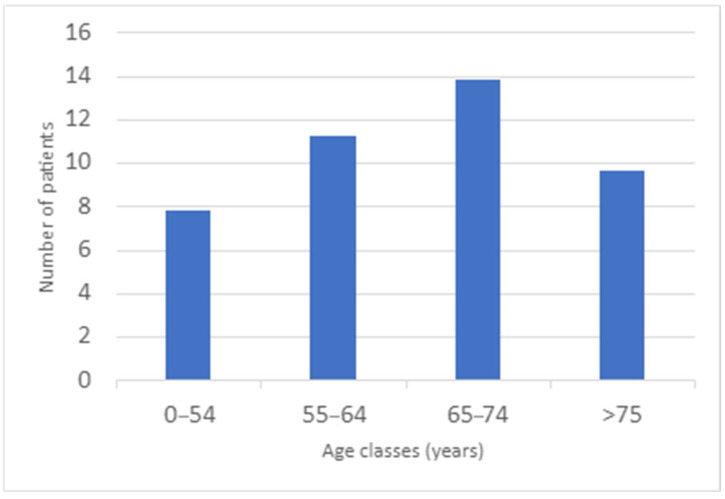
Prevalence of type 2 diabetes (T2D) in inflammatory bowel disease (IBD) patients.

A subdivision by age classes reveals a bell-shaped distribution of the association between inflammatory bowel diseases (IBDs) and type 2 diabetes (T2D) (Figure 3), with a marked peak in the 65–74 years old range (32.64%), followed by the 55–64 years old range (26.52%). This pattern suggests a possible age-dependent immunological interaction between these diseases, potentially due to the chronic inflammation seen in IBDs, which may escalate over time, influencing the development of insulin resistance and metabolic dysfunction, both of which are hallmarks of T2D.

The immune system’s pro-inflammatory environment in IBD, characterized by cytokine dysregulation (e.g., TNF-α, IL-6, IL-1β), may contribute to systemic inflammation, further exacerbating insulin resistance in older individuals. From a disease-specific perspective, ulcerative colitis (UC) appears to have a stronger correlation with T2D than Crohn’s disease (CD), particularly in the 65–74 years old range, followed by the 55–64 years old range. This may be attributed to the predominant Th2-driven immune response in UC, which is closely linked to systemic inflammation and metabolic disturbances. UC’s localized immune dysregulation, marked by T-helper cell activation and cytokine overproduction, may predispose these individuals to develop T2D at higher rates than patients with CD, who typically exhibit a more complex Th1/Th17-mediated immune response that may not have the same metabolic consequences. The gender distribution showed that 59.52% of the cohort were male, aligning with previous case-control studies. This supports the hypothesis that the association between T2D and IBDs (Figure 3) may be hypothetically sex-related, with males being more susceptible to this co-morbidity. Immunologically, males may have a different immune response to inflammatory stimuli, possibly influencing their risk of developing both IBDs and T2D. The cohort studied was characterized by a long duration of IBD (mean: 26.41 ± 11.25 years), with 73.81% of patients suffering from UC and 21.43% diagnosed with CD (4.76% had undetermined colitis). This highlights that, among the IBDs, UC is more strongly linked to T2D, which may be explained by UC’s more pronounced immune dysregulation and its role in driving systemic inflammation and insulin resistance. The chronic, low-grade intestinal inflammation seen in UC patients may be a key contributor to metabolic dysfunction, which aligns with increased risk for T2D. Analysis of body mass index (BMI) in the cohort showed that 35.71% of individuals were overweight, and 16.67% were obese, suggesting a positive correlation between BMI and IBDs.

**Figure 4:**
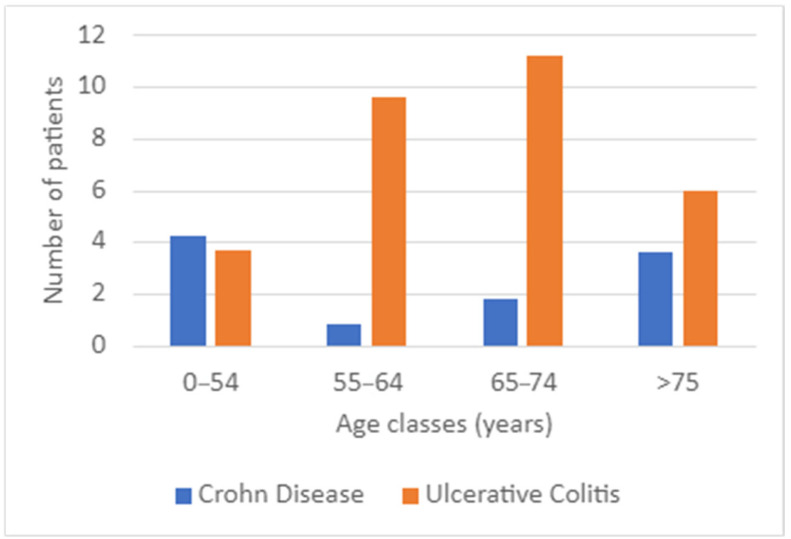
Inflammatory bowel disease (IBD) distribution in type 2 diabetes (T2D) patients.

From an immunological standpoint, increased BMI is known to enhance adipose tissue inflammation, which in turn amplifies systemic inflammation. This results in a vicious cycle that exacerbates both IBD and T2D (IBD distribution in T2D patients was shown in Figure 4).

Interestingly, within the higher BMI category (Table 3), overweight individuals (not obese) were more commonly linked to the coexistence of IBDs and DM (Table 4).

This might indicate that moderate obesity, with its characteristic low-grade inflammation, may contribute to both the pathogenesis of IBD and metabolic dysfunction seen in T2D. Additionally, family history of T2D was reported in 21.43% of the individuals, suggesting a genetic predisposition for both IBDs and diabetes. From an immunogenetic perspective, both conditions share certain genetic risk factors, such as polymorphisms in immune-related genes (e.g., *NOD2* for IBDs), which may increase susceptibility to both diseases. Furthermore, microbiome alterations are thought to play a key role in this relationship, with evidence pointing to changes in the gut microbiota, particularly at the phylum level, which could affect immune tolerance and promote chronic inflammation—key features in the pathogenesis of both IBDs and T2D [19]. A detailed evaluation of intestinal sites affected by IBDs in the cohort revealed that the transverse colon (38.09%) and sigmoid colon (26.19%) were most frequently affected (Table 5).

This distribution, although descriptive only, is of particular interest from an immunological standpoint, as these regions are often the sites of intense immune cell infiltration in UC, where T-lymphocytes (Thymus-derived lymphocytes) and macrophages play crucial roles in sustaining the inflammatory environment. Notably, in these sites, UC was the dominant pathology. Other areas, such as the ileum, large intestine, proximal rectum, and whole colon, showed more balanced involvement between UC and CD, indicating differing immunopathological mechanisms depending on the disease type.

## 4. Discussion

### 4.1. Case-Control Findings and Immunological Insights

The observed association between IBDs and DM, particularly T2D, reflects a complex interplay of immunological factors. The increased prevalence of DM among IBD patients, especially in younger age groups, may be related to the inflammatory pathways common to both diseases. This observation highlights the importance of understanding how systemic inflammation and immune dysregulation in IBD contribute to insulin resistance and metabolic disturbances. From an immunological perspective, the overactive Th2 and Th17 responses in UC are likely more prone to triggering insulin resistance, whereas the more heterogeneous immune profile in CD may not interact as directly with metabolic processes. The predominance of male patients and the association between IBD and T2D in this group could be explained by the immunological differences between sexes, with testosterone potentially playing a role in male susceptibility to inflammatory diseases. The finding that UC, more than CD, is linked with DM, as well as the strong association of T2D with immunosuppressive treatments, indicates that immune-modulating therapies may play a role in shaping the co-occurrence of these diseases. The use of immunosuppressive therapies in a significant portion of both IBD cases and controls further supports this theory, as these treatments can influence immune responses and disease outcomes. The higher rate of surgical intervention in IBD patients with DM emphasizes the need for careful management of both conditions, as DM can complicate surgical recovery and increase the risk of infections. Additionally, the immune dysregulation in IBD patients may contribute to systemic insulin resistance, which further predisposes them to developing T2D. The correlation between obesity and IBD, along with the presence of genetic predisposition, underscores the need for more targeted research into the genetic and environmental factors that influence the coexistence of these diseases. The potential genetic overlap between IBD and T2D raises interesting questions about shared susceptibility loci and the role of common immune-related pathways in driving the pathogenesis of both diseases. Finally, the limitations of this study, particularly the selection bias and lack of genetic profiling, highlight the need for further research to explore the genetic basis of the coexistence of IBD and DM. More studies incorporating genetic variability could provide deeper insights into the immunogenetic factors that drive the overlap between these two diseases.

### 4.2. Clinical and Immunological Cohort Insights

The findings from this study suggest a complex interplay between inflammatory bowel diseases (IBDs) and type 2 diabetes (T2D), with the association varying by age, disease type, gender, and BMI. The bell-shaped age distribution of IBD–T2D comorbidity, with peaks in the 65–74 and 55–64 age ranges, highlights the potential role of chronic inflammation in IBDs, which may escalate over time, contributing to metabolic dysfunction and insulin resistance typical of T2D. The pro-inflammatory cytokine environment in IBD, involving molecules like TNF-α, IL-6, and IL-1β, appears to exacerbate insulin resistance, especially in older individuals who may already be more vulnerable to metabolic disturbances. From a disease-specific standpoint, ulcerative colitis (UC) shows a stronger association with T2D than Crohn’s disease (CD), particularly in older age groups. This is likely due to the predominant Th2-driven immune response in UC, which is strongly linked to systemic inflammation and metabolic disturbances. UC’s immune dysregulation, marked by T-helper cell activation and cytokine overproduction, predisposes these individuals to develop insulin resistance and T2D at higher rates than those with CD, whose more complex Th1/Th17-driven immune response may not share the same metabolic consequences. The male predominance in the cohort further supports the hypothesis of a sex-related predisposition to both IBDs and T2D. Males may have a different immune response to inflammatory stimuli, which could contribute to their higher risk of developing both conditions. These findings align with previous studies showing that males are more susceptible to certain autoimmune diseases, including IBD. The long duration of IBD in the cohort (mean: 26.41 ± 11.25 years) and the higher prevalence of UC among patients with T2D suggest that chronic inflammation in UC could play a key role in the development of metabolic dysfunction. The chronic, low-grade intestinal inflammation seen in UC may not only drive gastrointestinal symptoms but also contribute to systemic inflammation, which is a key factor in the development of T2D. The chronic nature of UC may also explain its stronger correlation with T2D, as the prolonged immune activation may have more profound metabolic consequences. The significant correlation between BMI and IBD, especially within the overweight category, indicates that moderate obesity may contribute to the pathogenesis of both IBDs and T2D. Obesity is known to enhance adipose tissue inflammation, creating a vicious cycle that exacerbates both diseases. Additionally, the shared genetic predisposition for both IBDs and T2D, particularly through polymorphisms in immune-related genes like NOD2, further underscores the multifactorial nature of the connection between these two diseases. Alterations in the gut microbiota also appear to play a key role, with changes in microbiome composition potentially promoting inflammation and contributing to disease development. Finally, this study’s limitations, such as the potential selection bias due to the predominance of male patients and those in the 65–74 age group, may limit the generalizability of the findings. These demographic imbalances may also introduce confounding factors that influence disease progression and treatment outcomes. Future studies with more diverse and representative samples, as well as genetic profiling, will provide deeper insights into the immunological and genetic pathways that link IBDs and T2D, helping to refine therapeutic strategies and improve patient outcomes. Understanding the complex relationship between obesity, IBD, and T2D is crucial, as both conditions are driven by chronic inflammation and gut microbiota alterations. Obesity, particularly through visceral fat accumulation, enhances systemic inflammation, which can exacerbate IBD, especially in Crohn’s disease. Furthermore, the interplay between IBD and obesity-induced dysbiosis may create a self-reinforcing cycle that worsens both conditions. Targeting the microbiome, metabolism, and immune system could offer new therapeutic approaches for managing patients with co-occurring IBD and T2D [20,21].

### 4.3. UC–CD Correlation

Ulcerative colitis (UC) and Crohn’s disease (CD) are both inflammatory bowel diseases, but they involve distinct immune responses. UC is primarily associated with a Th2-dominated immune response, although Th17 cells can play a role in severe cases. Th2 cells produce cytokines like IL-4, IL-5, and IL-13, which lead to eosinophilic infiltration and mucosal inflammation in the colon. This inflammatory response, while mainly localized to the colon, can disrupt local metabolic regulation and immune responses. In contrast, CD is characterized by a Th1/Th17-dominant immune response, marked by cytokines like IL-12 and IL-17, which promote neutrophil and monocyte infiltration and the formation of granulomas. CD involves transmural inflammation that affects the entire thickness of the bowel wall and can impact any part of the gastrointestinal tract, particularly the ileum and colon. The Th2 response in UC causes mucosal inflammation with increased eosinophils and mast cells, which has systemic effects on gut-associated lymphoid tissues (GALT). Elevated levels of cytokines such as IL-5 and IL-13 contribute to tissue remodeling, fibrosis, and impaired wound healing. While IL-17 is more strongly associated with CD, recent studies suggest it also plays a role in UC, contributing to epithelial cell damage and promoting the release of IL-6 and other pro-inflammatory cytokines. In CD, Th17 cells are essential for granulomatous inflammation and intestinal barrier dysfunction, as IL-17 recruits neutrophils and monocytes, and disrupts epithelial tight junctions, leading to increased intestinal permeability. Although UC and CD have different immune profiles, both involve chronic systemic inflammation, which can predispose individuals to insulin resistance and type 2 diabetes (T2D). The pro-inflammatory cytokines released in both conditions, such as TNF-α, IL-6, and IL-1β, impair insulin receptor signaling, leading to insulin resistance. In UC, the Th2/Th17 immune response extends beyond the gut, with cytokines like IL-6, TNF-α, and IL-17 playing a role in the development of adiposity and insulin resistance. Visceral fat, in particular, produces pro-inflammatory cytokines that contribute to systemic inflammation and worsen insulin resistance. Both UC and CD are also associated with alterations in the gut microbiome, which can affect liver function and insulin sensitivity through the gut-liver axis. Dysbiosis in UC can increase intestinal permeability, allowing microbial products to enter the bloodstream and promote systemic inflammation, further impairing insulin sensitivity.

The chronic inflammation in UC, driven by cytokines like IL-4, IL-13, and IL-17, leads to systemic inflammation that can impair insulin signaling and contribute to insulin resistance in peripheral tissues such as muscle, liver, and adipose tissue. UC patients also often experience weight loss and malnutrition, which further disrupt metabolism and predispose them to T2D. Chronic inflammation in adipose tissue releases pro-inflammatory adipokines, such as resistin and leptin, which exacerbate insulin resistance.

The immunological mechanisms underlying UC and CD, especially the roles of Th2 and Th17 responses, not only drive intestinal inflammation but also contribute to systemic inflammation, which can predispose individuals to T2D. A better understanding of the shared cytokine profiles, adipose tissue dysfunction, and gut–liver interactions between UC and T2D can provide deeper insights into the mechanisms linking chronic inflammation in IBD to metabolic diseases.

### 4.4. Pathophysiology

Existing findings, such as the observed relationship between ulcerative colitis (UC) and increased surgery rates in patients with elevated body mass index (BMI), warrant deeper exploration within a pathophysiological framework. Obesity is characterized by chronic low-grade inflammation and altered immune responses, which may exacerbate the severity of inflammatory bowel disease (IBD) and complicate surgical outcomes. In particular, increased visceral adiposity is associated with heightened levels of pro-inflammatory cytokines like tumor necrosis factor-alpha (TNF-α) and interleukin-6 (IL-6), which are also key mediators in the pathogenesis of UC. These cytokines contribute not only to persistent intestinal inflammation but also to systemic insulin resistance, thereby offering a mechanistic link between UC and type 2 diabetes (T2D) [22]. The pathophysiological intersection between UC and T2D appears to be grounded in shared immune dysregulation and systemic inflammation. In UC, aberrant mucosal immune responses to commensal gut microbiota lead to intestinal inflammation, compromised epithelial barrier function, and increased intestinal permeability—a phenomenon often referred to as “leaky gut” [23]. This disrupted barrier permits translocation of microbial products such as lipopolysaccharides (LPS) into the systemic circulation, promoting metabolic endotoxemia, which has been implicated in insulin resistance and the development of T2D [24].

While this study provides valuable insights, it demonstrates insufficient control for potential confounding factors, with no adjustments made for variables such as age and sex. These factors can influence the observed outcomes, and their omission may introduce bias or limit the generalizability of the results. However, the findings remain informative within the context of this study’s design and should be interpreted with these limitations in mind. Furthermore, T-cell dysregulation—particularly a shift toward pro-inflammatory Th17 cells and a reduction in regulatory T-cells (Tregs)—is a hallmark of both UC and T2D, underscoring a common immunological axis. Interestingly, epidemiological data suggest that UC, more so than Crohn’s disease (CD), shows a stronger association with T2D, particularly among older adults aged 65–74 years. This age-specific risk elevation may reflect immunosenescence and inflammaging—age-related changes in immune function and chronic inflammation—that further impair metabolic and intestinal homeostasis.

Additionally, alterations in gut microbiota composition (dysbiosis) seen in UC patients are known to influence host metabolism, insulin sensitivity, and systemic inflammatory status. Reduced microbial diversity and a decline in short-chain fatty acid–producing bacteria may contribute to both intestinal inflammation and metabolic dysfunction, further reinforcing the UC–T2D connection [25]. Taken together, these insights highlight the intricate immunometabolic crosstalk underlying UC and T2D, suggesting that integrated management strategies addressing both gastrointestinal and metabolic inflammation are essential [26,27,28,29,30,31,32]. Therapeutic approaches targeting shared pathways—such as cytokine inhibition, microbiome modulation, and enhancement of epithelial barrier function—could offer dual benefits for patients affected by both conditions.

## 5. Conclusions

The relationship between inflammatory bowel diseases (IBD) and type 2 diabetes (T2D) is increasingly recognized as clinically significant, with emerging evidence suggesting a meaningful association—particularly in cases of ulcerative colitis (UC). Findings from this study indicate that patients with IBD, especially UC, may be at higher risk for developing T2D. This comorbidity appears to be more pronounced when UC is localized in the transverse and sigmoid regions of the colon, suggesting that disease location may influence systemic metabolic outcomes. One possible mechanism underpinning this association is chronic inflammation, which is a hallmark of both IBD and T2D. It is hypothesized that sustained inflammatory responses—characterized by elevated levels of biomarkers such as C-reactive protein (CRP), interleukin-6 (IL-6), and tumor necrosis factor-alpha (TNF-α)—may contribute to the development of insulin resistance, a central feature in the pathogenesis of T2D. Therefore, inflammation may act as a shared pathophysiological pathway linking these two seemingly distinct conditions. Additionally, this study reinforces the observation that IBD patients tend to exhibit higher body mass index (BMI), which is itself a well-established risk factor for T2D. This association further supports the notion of overlapping risk profiles and mechanisms, as both IBD and T2D are influenced by a combination of genetic predisposition and environmental factors, including diet, microbiome alterations, and lifestyle.

However, the strength of these conclusions is tempered by certain limitations, most notably the relatively small sample size—48 cases and 96 controls in the cross-sectional component, and only 49 cases in the cohort study. Such limited numbers reduce statistical power and may restrict the generalizability of the findings to broader and more diverse populations. As such, while the associations observed are compelling, they must be interpreted with caution.

Looking ahead, future research should prioritize large-scale, longitudinal studies to clarify the temporal and causal relationships between IBD and T2D. Special attention should be given to tracking inflammatory biomarkers over time in IBD patients to assess their predictive value for insulin resistance. Moreover, genetic and molecular studies could provide further insight into shared pathways, while investigations into the metabolic effects of anti-inflammatory therapies may offer therapeutic implications.

In conclusion, while this study strengthens the evidence for an association between IBD—particularly UC—and T2D, further multidisciplinary research is essential. Establishing chronic inflammation as the central mechanistic link could ultimately reshape clinical approaches to managing patients with IBD, emphasizing early metabolic screening and integrated treatment strategies aimed at reducing long-term complications such as diabetes.

## Figures and Tables

**Table 1:** Case-control patients demographics, IBD type, comorbidities and BMI. SD: standard deviation. BMI: body mass index.

Parameters	Mean ± SD	Number (%)
Sex	-	
Males		30 (62.5%)
Females		18 (37.5%)
Age (years)	65.1 ± 11.8	
Disease type	-	
Ulcerative colitis		34 (70.8%)
Crohn’s disease		12 (25.0%)
Undetermined colitis		2 (4.2%)
Disease duration (years)	28.3 ± 9.4	
BMI (kg/m^2^)—mean value	27.4 ± 4.2	
BMI	-	
Overweight (25–30)		19 (39.6%)
Obesity (>30)		8 (16.7%)
Diabetes mellitus familiarity	-	15 (31.3%)
Diabetes type	-	
Type 1		9 (18.7%)
Type 2		39 (81.3%)

**Table 2:** Case-control patients therapeutic regimens.

Therapy	Cases (*n* = 48)	Controls (*n* = 96)	*p* Values
Immunosuppressive treatment	13 (27.1%)	20 (20.8%)	n.s.
Biological treatment	2 (4.2%)	13 (13.5%)	n.s.
Surgery	10 (20.8%)	7 (7.2%)	0.036
Ileal Pouch	4 (11.7%)	3 (4.4%)	0.10

Note: n.s. (non significant).

**Table 3:** Case-control patients surgery necessity rates, subdivided according to BMI, diabetes type, therapy and diabetes onset. BMI: body mass index. IBD: inflammatory bowel disease.

Parameters	Surgery Necessity		*p* Values
	Yes (*n* = 10)	No (*n* = 38)	
BMI			1.000
Normal weight < 25	4	17	
Overweight > 25	6	21	
Diabetes type			1.000
Type 1	3	6	
Type 2	7	32	
Therapy			0.238
Insulin	1	14	
Oral hypoglycemizing drugs	9	24	
Diabetes onset			0.663
Before IBD	1	9	
After IBD	9	29	

**Table 4:** Cohort study’s patients demographics, IBD type, comorbidities and BMI. SD: standard deviation. BMI: body mass index.

Parameters	Mean ± SD	Number (%)
Sex	-	
Males		25 (59.52%)
Females		17 (40.43%)
Age	63.55 ± 11.56	-
Disease type	-	
Ulcerative colitis		31 (73.81%)
Crohn’s disease		9 (21.43%)
Undetermined colitis		2 (4.76%)
Disease duration (years)	26.41 ± 11.25	-
BMI (kg/m^2^)—mean value	22.71 ± 4.44	-
BMI	-	
Overweight (25–30)		15 (35.71%)
Obesity (>30)		7 (16.67%)
Diabetes familiarity	-	
Type 1		4 (9.52%)
Type 2		9 (21.43%)

**Table 5:** Cohort study’s patients IBDs location in gut. UC: ulcerative colitis, CD: Crohn’s disease, NDC: non-determined colitis.

IBD Type	Transverse Colon	Sigmoid Colon	Ileus	Large Intestine	Proximal Rectum	Whole Colon	Total
UC	35.71	21.43	0	0	11.90	4.76	73.81
CD	2.38	0	7.14	9.52	0	2.38	21.43
NDC	0	4.76	0	0	0	0	4.76
Total	38.09	26.19	7.14	9.52	11.90	7.14	100

## Data Availability

The data used to support the findings of this study are available from the corresponding authors upon request.

## Abbreviations

ADA	American Diabetes Association
BMI	Body Mass Index
CD	Crohn’s Disease
DM	Diabetes Mellitus
FPG	Fasting Plasma Glucose
IBD	Inflammatory Bowel Disease
IL-6	Interleukin-6
IL-1β	Interleukin-1 Beta
NDC	Non-Determined Colitis
NOD2	Nucleotide-binding Oligomerization Domain-containing protein 2
NSAIDs	Nonsteroidal Anti-Inflammatory Drugs
n.s.	Non-Significant
OGTT	Glucose Tolerance Test
T1D	Type 1 Diabetes
T2D	Type 2 Diabetes
TNF-α	Tumor Necrosis Factor-alpha
UC	Ulcerative Colitis
